# Rare Metastasis of Gastric Cancer to the Thyroid Gland: A Case Report and Review of Literature

**DOI:** 10.3389/fsurg.2021.731673

**Published:** 2021-10-08

**Authors:** Zehui Wu, Tao Guo, Qiang Li, Liang Cheng, Xiaosi Hu, Aman Xu

**Affiliations:** ^1^Department of General Surgery, The First Affiliated Hospital of Anhui Medical University, Hefei, China; ^2^Department of Gastrointestinal Surgery, The First Affiliated Hospital of Wannan Medical College, Wuhu, China; ^3^Department of General Surgery, The Fourth Affiliated Hospital of Anhui Medical University, Hefei, China; ^4^Department of Medical Records, The First Affiliated Hospital of Wannan Medical College, Wuhu, China; ^5^Department of Radiology, The First Affiliated Hospital of Wannan Medical College, Wuhu, China

**Keywords:** gastric cancer, metastasis, thyroid, diagnosis, prognosis, case report

## Abstract

**Background:** It is common for patients with gastric cancer to develop distant metastases in the liver, lung, bone, and brain. Although the thyroid also has an abundant blood supply, gastric cancer metastasis to the thyroid is uncommon. Due to the rarity of such metastasis, its clinical features are not well understood. Here, we present the case of a patient with gastric cancer metastasis to the thyroid treated at our hospital.

**Case Summary:** We report the case of a 63-year-old female with a mass in the anterior neck and mild hoarseness for 6 months. The patient underwent proximal subtotal gastrectomy for Siewert III oesophagogastric junction cancer 6 years ago. Subsequently, she received 8 cycles of adjuvant chemotherapy. Her condition was stable until mild hoarseness developed for no apparent reason 6 months prior to presenting at our clinic. Both ultrasonography and computed tomography confirmed a heterogeneous mass in the right lobe of the thyroid gland. Blood thyroid function tests and tumor marker expression levels were normal. Thyroid malignancy was suspected, and the patient underwent a right thyroidectomy. During the surgery, a tumor was found that had invaded the right recurrent laryngeal nerve and trachea. H&E staining and immunohistochemistry results suggested that the cancer cells originated from gastric cancer. The patient was diagnosed with thyroid metastasis of gastric cancer. She refused further treatment and died within 6 months.

**Conclusion:** Metastasis of gastric cancer to the thyroid is rare and is associated with a poor prognosis. Immunohistochemical diagnosis is essential for a conclusive diagnosis. For patients with a history of malignant tumors, the possibility of metastatic thyroid nodules should be ruled out when diagnosing thyroid nodules.

## Introduction

Lymph node metastasis and haematogenous metastasis are the main metastasis pathways of gastric cancer. It is common for patients with gastric cancer to develop distant metastases in the liver, lung, bone, and brain. Although the thyroid also has an abundant blood supply, gastric cancer metastasis to the thyroid is uncommon. To the best of our knowledge, only 13 cases of thyroid metastasis of gastric cancer have been reported in the English literature. Due to the rarity of this condition, its clinical features are not well understood. Here, we present the case of a patient with gastric cancer metastasis to the thyroid treated at our hospital.

## Case Presentation

### Clinical History and Findings

A 63-year-old female with an anterior neck mass and mild hoarseness sought treatment at our clinic. The patient's condition was stable until mild hoarseness developed for no apparent reason 6 months prior to presentation at our clinic. However, the patient was not concerned by this symptom. Subsequently, the patient found a painless mass on her anterior neck. There were no other clinical symptoms, such as dyspnoea, neck pain, nervousness or rapid heartbeat. Six years prior, the patient underwent a proximal subtotal gastrectomy and lymph node dissection for Siewert III oesophagogastric junction cancer. The histologic type was moderately differentiated adenocarcinoma with ulceration. The cancer had grown into the serosa of the stomach (T3). Cancer cells were found in 8 nearby lymph nodes (N3), but no distant metastasis was observed. Therefore, the patient's disease was classified as T3N3M0, stage IIIb. Eight cycles of chemotherapy (5-FU and cisplatin) every 3 weeks were administered after surgery. The patient's condition remained stable each year during the follow-up period. No other personal or family history of disease was reported. Physical examination at admission revealed a solitary painless thyroid nodule, 2 cm by 3 cm, in the right lobe of the thyroid gland that moved up and down with swallowing. Tests revealed that the full blood count, liver function, renal function, thyroid function, and level of serum tumor markers, such as carcinoembryonic antigen (CEA), carbohydrate antigen 19–9 (CA19-9), carbohydrate antigen 125 (CA125) and alpha-fetoprotein (AFP) were within normal ranges.

### Diagnostic Assessment

**Figure 1:**
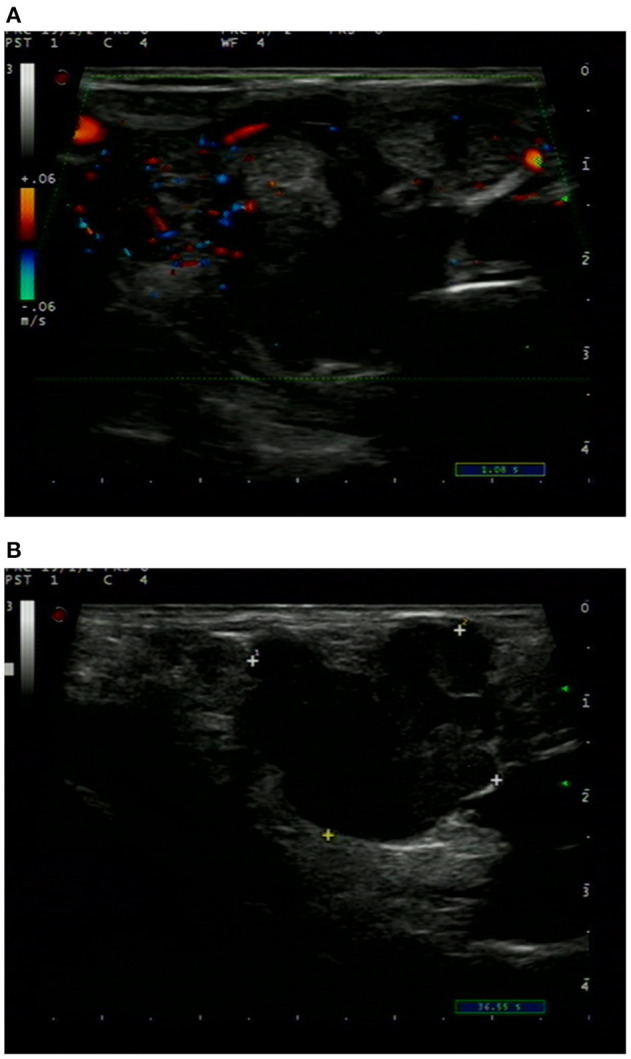
Ultrasound sonogram of the thyroid showed a non-homogenous mass with an unclear boundary, low and moderate echo and rich blood flow signal **(A)**. Enlarged lymph nodes were found in the right neck **(B)**.

**Figure 2:**
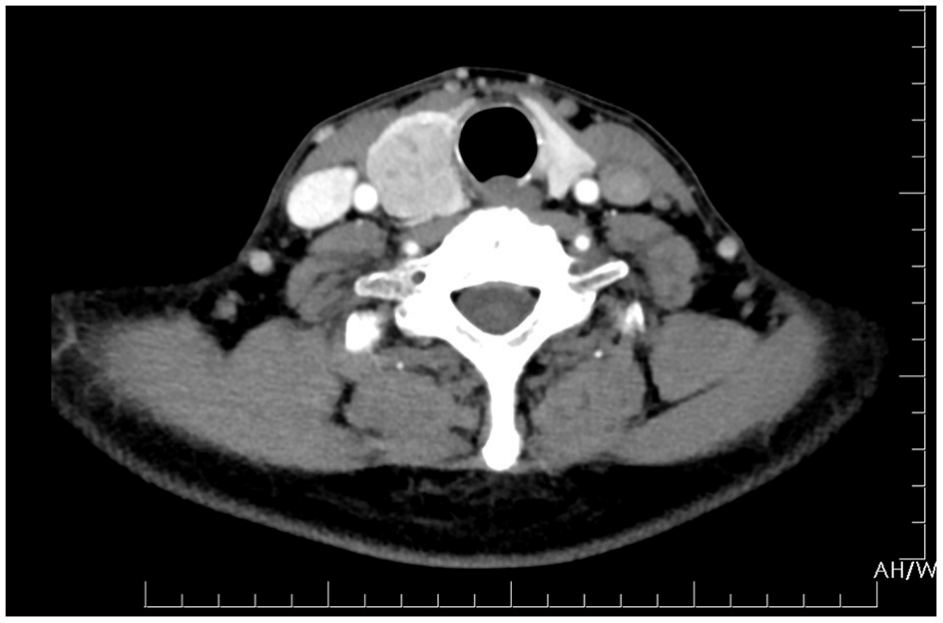
CT of the neck showed a tumor occupying almost the entire right lobe of the thyroid gland.

Ultrasonography revealed a non-homogenous hypoechoic solid mass, 49 mm × 34 cm in size, with blurred boundaries and a rich blood flow signal, in the right lobe of the thyroid gland (Figure 1A). Several enlarged lymph nodes were found in the right neck (Figure 1B). Computed tomography (CT) revealed that the tumor occupied almost the entire right lobe of the thyroid gland, with heterogeneous enhancement on the arterial phase (Figure 2). Direct laryngoscopy showed that the appearance and movement of the left vocal cord appeared normal, while the right vocal cord had some dyskinesia. Given these results, a malignant tumor of the thyroid was suspected. Fine needle aspiration was suggested, but the patient refused this procedure and insisted on thyroid surgery.

### Treatment and Diagnosis

**Figure 3:**
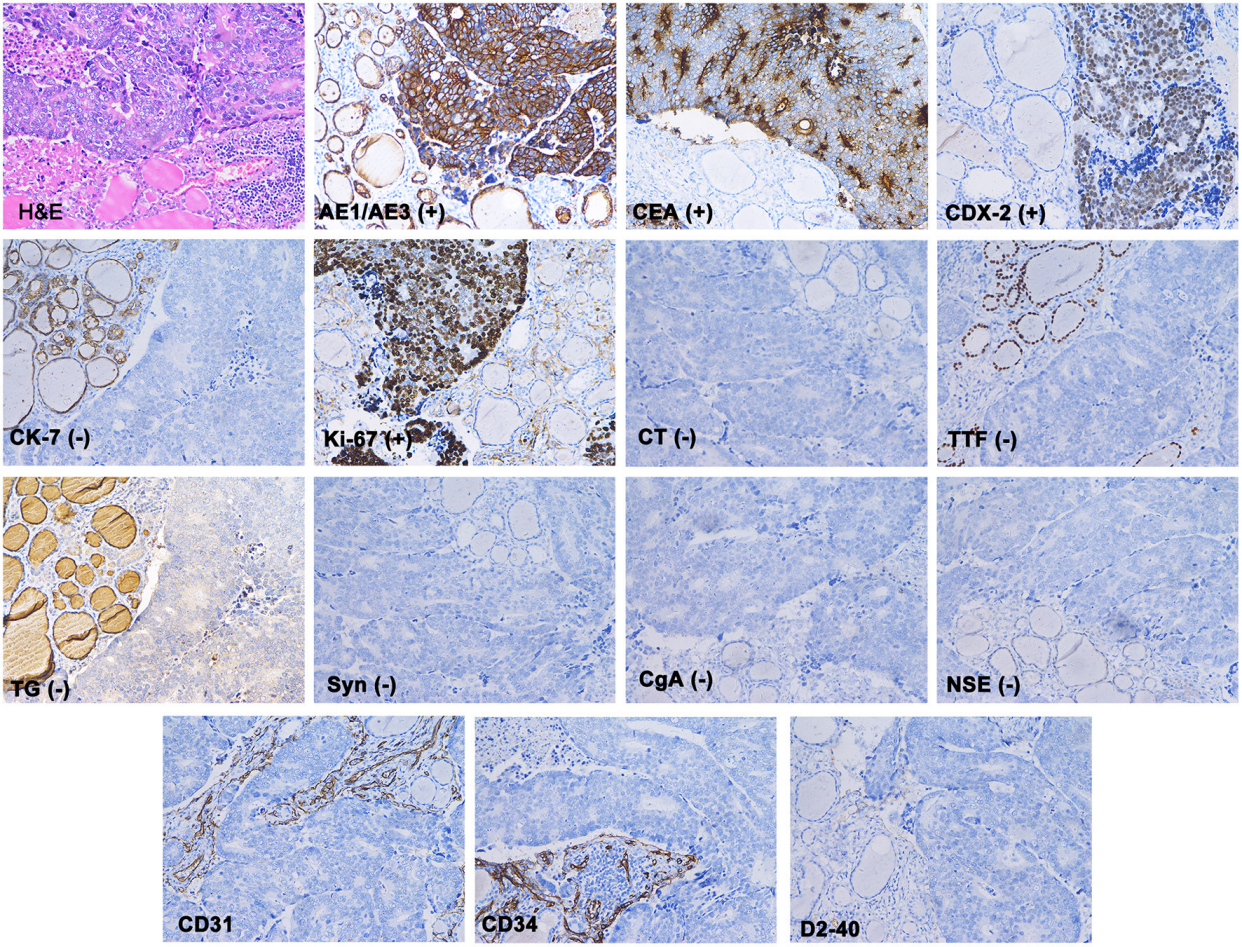
Haematoxylin and eosin staining showed that the normal thyroid follicles had been destroyed by dispersed tumor cells. Immunohistochemistry showed that AE1/AE3, CEA and CDX-2 stained positive, while CK7, TG, TTF, CT, Syn, CgA, and NSE were absent. The blood vessels (marked with CD31 and CD34) and lymphatic vessels (marked with D2-40) were found to be filled with tumor cells.

The patient underwent a right thyroidectomy, and the tumor was found to have invaded the right recurrent laryngeal nerve and trachea. Intraoperative frozen section analysis showed that the thyroid follicles had been diffusely infiltrated by poorly differentiated tumor cells with morphology resembling gastrointestinal lesions. The blood vessels and lymphatic vessels were filled with tumor cells. The following immunohistochemistry staining results were obtained (Figure 3): AE1/AE3 (+), CEA (+), CDX-2 (+), cytokeratin 7 (–), Ki-67 90% (+), calcitonin (–), thyroid transcription factor-1 (–), thyroglobulin (–), synaptophysin (–), chromogranin A (–), and neuron-specific enolase (–). The characteristics of these tumors more closely resembled those of metastatic gastric adenocarcinoma than primary thyroid malignancies. Based on the patient's history of gastric cancer, the histopathological evaluation and immunohistochemistry staining results, the patient was diagnosed with gastric cancer metastasis to the thyroid.

### Outcome and Follow-Up

There were no complications with the surgery. However, the patient refused further treatment and died from general deterioration within 6 months.

## Discussion

The thyroid gland is a relatively infrequent site of metastasis. Although some autopsy studies have indicated that thyroid metastasis is more common than previously believed, clinically evident thyroid masses remain rare. Approximately 0.05–0.3% of thyroid malignancies excised at various centers are metastatic tumors (1, 2). The most common primary cancer sites are the kidney, breast, colon and lung (3–7). Cases of thyroid-metastatic tumors originating from the stomach are rare. To the best of our knowledge, only 13 cases of thyroid metastasis of gastric cancer have been reported in the PubMed-indexed English literature (7–16). Our article presents an additional case in a Chinese woman and reviews the relevant literature.

The clinical data for the 13 cases previously published, plus our case of a thyroid-metastatic tumor from gastric adenocarcinoma, are summarized in Table 1. Patient age at presentation with thyroid metastasis ranged between 39 and 71 years (median = 62.5 years), and the male to female ratio was 3:4. The majority of primary gastric cancers were poorly differentiated adenocarcinoma or signet-ring carcinoma (specific types are presented in Table 1). Synchronous thyroid metastasis was found in nine cases (7, 11–13, 15, 16), and four of these patients also had metastases in other organs at the time of diagnosis (7, 11, 12, 15). Metachronous metastases occurred in the other five cases (8–10, 14). The interval between the initial diagnosis of the primary tumor and that of metastasis ranged from 3–70 months. In the current study, a latency period of 70 months was the longest of all reported cases. Among 10 patients with available survival data, only one patient survived more than 1 year. Nine patients lived 1 year or less, and eight lived 6 months or less.

**Table 1:** Clinical data of patients with metastatic thyroid tumors from gastric cancer.

**Case no. (reference)**	**Sex**	**Age (years)**	**Primary pathology**	**Primary treatment**	**Interval (months)**	**Metastatic cancer pathology**	**Metastatic cancer treatment**	Survival (months)^*^
1 (7)	F	39	Adenocarcinoma	None	0	Adenocarcinoma	None	1
2 (8)	M	71	Poorly	Total gastrectomy	60	Poorly	Subtotal thyroidectomy	11
2 (9)	F	60	Poorly	Subotal gastrectomy	3	Poorly	Bilateral subtotal thyroidectomy	1
4 (10)	F	63	Poorly	Distal subotal gastrectomy	14	Signet-ring	Chemotherapy	6
5 (11)	M	71	Poorly	None	0	Poorly	Bilateral near-total thyroidectomy	4
6 (12)	M	68	Signet-ring	None	0	Signet-ring	None	1
7 (13)	M	67	Signet-ring	Gastrectomy	0	Signet-ring	Thyroidectomy + chemotherapy	Alive (14 months)
8 (14)	M	58	Poorly	Subtotal gastrectomy	35	Poorly	Radiotherapy	5
9 (15)	F	74	Poorly	None	0	Poorly	Chemotherapy	1
10 (16)	F	50	Adenocarcinoma	NA	0	Adenocarcinoma	NA	NA
11 (16)	F	62	Adenocarcinoma	NA	0	Adenocarcinoma	NA	NA
12 (16)	M	41	Adenocarcinoma	NA	0	Adenocarcinoma	NA	NA
13 (16)	F	46	Adenocarcinoma	NA	0	Adenocarcinoma	NA	NA
Present case	F	63	Moderate	Proximal subtotal gastrectomy	70	poorly	None	6

* *Follow up since diagnosis of intrathyroid metastases; poorly, poorly differentiated adenocarcinoma; moderate, moderate differentiated adenocarcinoma; signet-ring, signet-ring carcinoma; NA, no data available*.

Ultrasonography (US) is a widely used, non-invasive method for risk stratification of thyroid nodules. By using US, we can easily determine whether a thyroid nodule is solitary or multiple, as well as its size, morphology, and blood supply (17). US is sensitive but not specific. Primary thyroid malignancies with papillary or follicular histology are difficult to distinguish from secondary cancers of other organs (18). Thyroid = metastatic tumors from gastric cancer can present as solid or diffuse masses. In most reports, the tumors were heterogeneous (12–14). However, these features are not unique. Contrast-enhanced CT scans can provide detailed anatomic information and can detect more lymph node metastasis than US alone (19, 20). Combining CT with US improves the detection of the morphological and blood supply characteristics of thyroid nodules. Nevertheless, it cannot identify the tumor's origin when a malignant tumor is suspected.

Fine needle aspiration cytology (FNAC) can assist in the differentiation between benign and malignant thyroid diseases (21). Although we did not perform FNAC, we believe that ultrasound-guided FNAC is the most accurate and cost-effective test for evaluating thyroid nodules in current practice (22). The success rate of thyroid FNAC is influenced by many factors, such as the nature of the lesions, the experience of the operator and the rational analysis of cytological samples. In the previous clinical cases, 12 patients were diagnosed with malignant thyroid nodules based on FNAC (7, 8, 10–16). Initially, the shape and morphology of thyroid tumor cells must be observed. The presence of signet ring cells is unusual and should be considered indicative of potential thyroid metastasis. However, it should be noted that signet ring cells can also appear in some primary thyroid tumors, such as signet ring cell follicular adenoma, which is characterized by signet ring cells with positive thyroglobulin staining (23, 24). Various molecular markers should be immunohistochemically evaluated to further determine the origin of the cancer cells. The main immune markers used to differentiate thyroid-metastatic carcinoma from primary thyroid carcinoma are thyroglobulin (TG), thyroid transcription factor 1 (TTF-1), Cdx-2, CK7, and CK20 (6, 25). Primary lesions are typically TG and TTF-1-positive, whereas metastatic lesions are typically negative for these markers. However, ~20–30% of primary anaplastic thyroid carcinomas are also negative for the above markers (26). Cdx-2, CK7, and CK20 are expressed in the gastrointestinal epithelium and are widely used in the diagnosis of metastatic carcinoma of the digestive tract (27). Medullary thyroid carcinoma markers include calcitonin (CT), chromogranin A (CgA), and synaptophysin (Syn). Immunohistochemistry, in our case, revealed that the tumor cells were positive for AE1/AE3, CEA, and CDX-2 and negative for CK7, TG, TTF, CT, Syn, CgA, and NSE. These results are consistent with those of previous studies and strongly suggest that the tumor originated from the digestive tract rather than the primary thyroid gland.

Compared with the rate of metastasis to other organs, the thyroid is a rare metastasis site, despite its abundant blood supply. Two hypotheses have been proposed by Willis (28): (1) thyroid glands have a rich blood supply and fast blood flow, which may prevent cancer cell colonization, and (2) the high iodine- and oxygen-rich environment in thyroid tissue may inhibit the proliferation of cancer cells. Therefore, it is generally believed that cancer cell survival is possible only when thyroid adenomas or inflammation cause changes in local tissue microenvironments. This raises the question of how gastric cancer can metastasize to the thyroid and whether this process occurs via the haematogenous pathway, the lymphatic pathway, or both. The answer to this question is up for debate. In our case, we stained blood vessels and lymphatic vessels with CD31, CD34, D2-40, and found abundant cancer cells in both sets of vessels. Therefore, in addition to haematogenous metastasis, there is a possibility of lymphatic metastasis in this case.

In patients with thyroid metastasis, surgical resection may be considered if their physical condition is good. However, the extent to which resection prolongs survival is still controversial because thyroid metastasis often coincides with generalized metastasis. We suggest that the decision to perform surgery should be made considering the level of progression of the primary disease. For the detection of metastatic disease, PET-CT scans are an extremely useful diagnostic tool (29). In many cases, intrathyroid metastatic tumors are themselves discovered *via* PET-CT (15, 30–33). If the primary tumor can be completely removed in the absence of other organ metastases or if the metastases can be removed simultaneously, the surgical treatment of thyroid metastases should be considered. In cases of unresectable primary tumors, multiple metastases, and no obvious tracheal compression, chemotherapy, instead of surgery, should be considered. If the location of the primary tumor cannot be determined, resection of metastatic thyroid cancer tissue can help to locate the primary tumor.

## Conclusion

We conclude that thyroid metastasis of gastric cancer is rare and is associated with a poor prognosis. Immunohistochemical analysis is essential for a conclusive diagnosis. For patients with a history of malignant tumors, the possibility of metastatic thyroid nodules should be ruled out when diagnosing thyroid nodules.

## Data Availability Statement

The original contributions presented in the study are included in the article/supplementary material, further inquiries can be directed to the corresponding author.

## Ethics Statement

Written informed consent was obtained from the individual(s) for the publication of any potentially identifiable images or data included in this article.

## Author Contributions

AX: guarantees the integrity of the entire study and edited the manuscript. ZW and TG: prepared and edited the manuscript. QL, LC, and XH: performed the literature research, data analysis, and text proofreading. All authors contributed to the article and approved the submitted version.

## Funding

This study was supported by grants from the Basic and Clinical Cooperative Research and Promotion Program of Anhui Medical University (2020xkjT045) and the National and Provincial Key Specialty Construction Plan (No. Z155080000004).

## Conflict of Interest

The authors declare that the research was conducted in the absence of any commercial or financial relationships that could be construed as a potential conflict of interest.

## Publisher's Note

All claims expressed in this article are solely those of the authors and do not necessarily represent those of their affiliated organizations, or those of the publisher, the editors and the reviewers. Any product that may be evaluated in this article, or claim that may be made by its manufacturer, is not guaranteed or endorsed by the publisher.
